# 2023 *BMC Ecology and Evolution* image competition: the winning images

**DOI:** 10.1186/s12862-023-02141-x

**Published:** 2023-08-18

**Authors:** Jennifer Harman, Christy A. Hipsley, Luke M. Jacobus, David A. Liberles, Josef Settele, Arne Traulsen

**Affiliations:** 1 BMC, London, UK; 2 University of Copenhagen, Copenhagen, Denmark; 3 Indiana University-Purdue University Columbus, (IUPUC), Columbus, IN USA; 4 Temple University, Pennsylvania, USA; 5 Helmholtz-Centre for Environmental Research – UFZ, Leipzig, Germany; 6 Max-Planck Institute for Evolutionary Biology, Schleswig-Holstein, Germany

## Abstract

In 2023, researchers from around the world entered the *BMC Ecology and Evolution* photography competition. As a result, we received a spectacular collection of photographs that capture the wonder of nature, those looking to understand it and glimpses into long lost worlds. This editorial celebrates the winning images selected by the Editor of *BMC Ecology and Evolution* and senior members of the journal’s editorial board.

## Introduction

We are thrilled to announce the winning images of our annual *BMC Ecology and Evolution* photography competition. Like in previous years [1–9], we received a spectacular collection of images from ecologists and evolutionary biologists from around the globe. *BMC Ecology and Evolution* invited anyone affiliated with a research institution to submit to one of the following four categories: ‘Research in Action’, ‘Protecting our planet’, ‘Plants and Fungi’ and ‘Paleoecology’.

Our Senior Editorial Board Members lent their expertise to judge the submissions, selecting the overall winner, best image, and runner-up from each category. The board members considered the scientific story behind the photos in addition to their artistic judgement.

## Overall winner

**Fig. 1:**
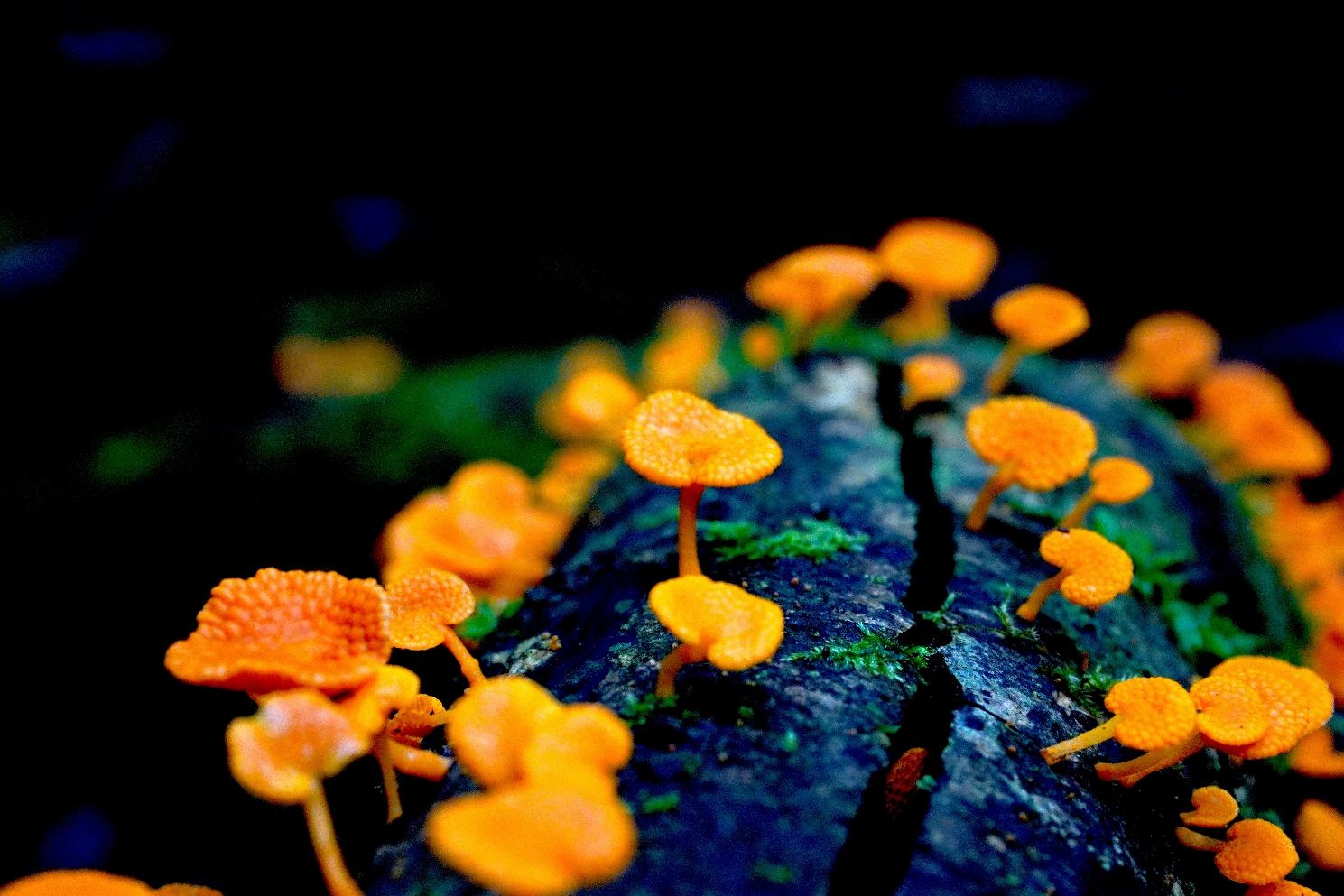
An invasive orange pore fungus poses unknown ecological consequences for Australian ecosytems. Attribution: Cornelia Sattler

The overall winner depicts the fruiting bodies of the invasive orange pore fungus (*Favolaschia calocera*), which poses unknown ecological consequences. While fungi play crucial roles in maintaining ecological balance, this visually striking species is causing concern. Photographer Cornelia Sattler comments, “Despite its innocent and beautiful appearance, the orange pore fungus is an invasive species in Australia. This species is displacing other fungi and spreading throughout the Australian rainforest. The bright orange fruiting bodies typically grow on deadwood and can spread through spores, often transported by humans.“ The ramifications of this invasive species on Australian ecosystems remain uncertain. It is important to closely monitor and understand the impact of this invasive fungus to mitigate any unforeseen consequences and safeguard the biodiversity of Australia. Senior Editorial Board Member Arne Traulsen comments the photo “allows a peek into a world that seems extremely different from ours. Fungi are fascinating but seem underappreciated and understudied”. (Fig. 1).

## Research in action: best in category

**Fig. 2:**
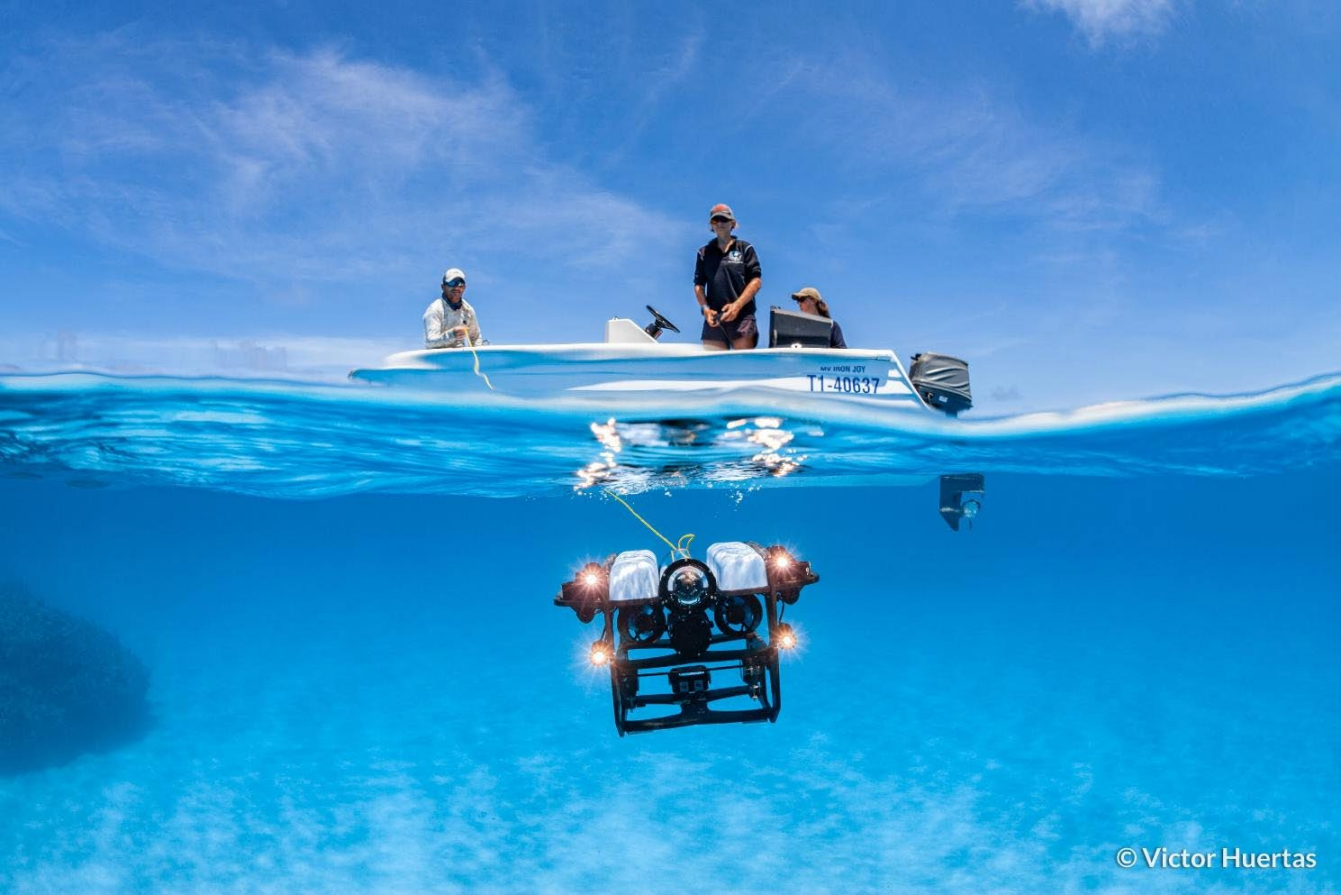
Exploring the deep. Researchers from the Hoey Reef Ecology Lab deploy an underwater ROV at Diamond Reef within the Coral Sea Marine Park. Attribution: Victor Huertas

Victor Huertas, a Postdoctoral Research Associate from the Hoey Reef Ecology Lab at James Cook University in Australia captured the winning image for the ‘Research in action’ category. The photo beautifully captures a moment as the team deploys an underwater Remotely Operated Vehicle (ROV) at Diamond Reef within the Coral Sea Marine Park. This advanced ROV, equipped with multiple photo and video cameras, serves as a vital tool enabling surveys at depths beyond the reach of divers. Thanks to these devices, the team has uncovered new species in reefs where they had not yet been documented, expanding the geographic range of multiple fish species. This achievement exemplifies the exciting possibilities that arise from the development and accessibility of new technologies in field studies, pushing the boundaries of scientific knowledge and enriching our understanding of life underwater. Senior Editorial Board Member Luke Jacobus comments “This photograph captures the essence of ecological study. It showcases sharp imaging and good storytelling as we see humans acting at the interface of the atmosphere and the hydrosphere. The motion of the water and the scientific device invite us to be curious about our dynamic world.” (Fig. 2).

## Research in action: runner-up

**Fig. 3:**
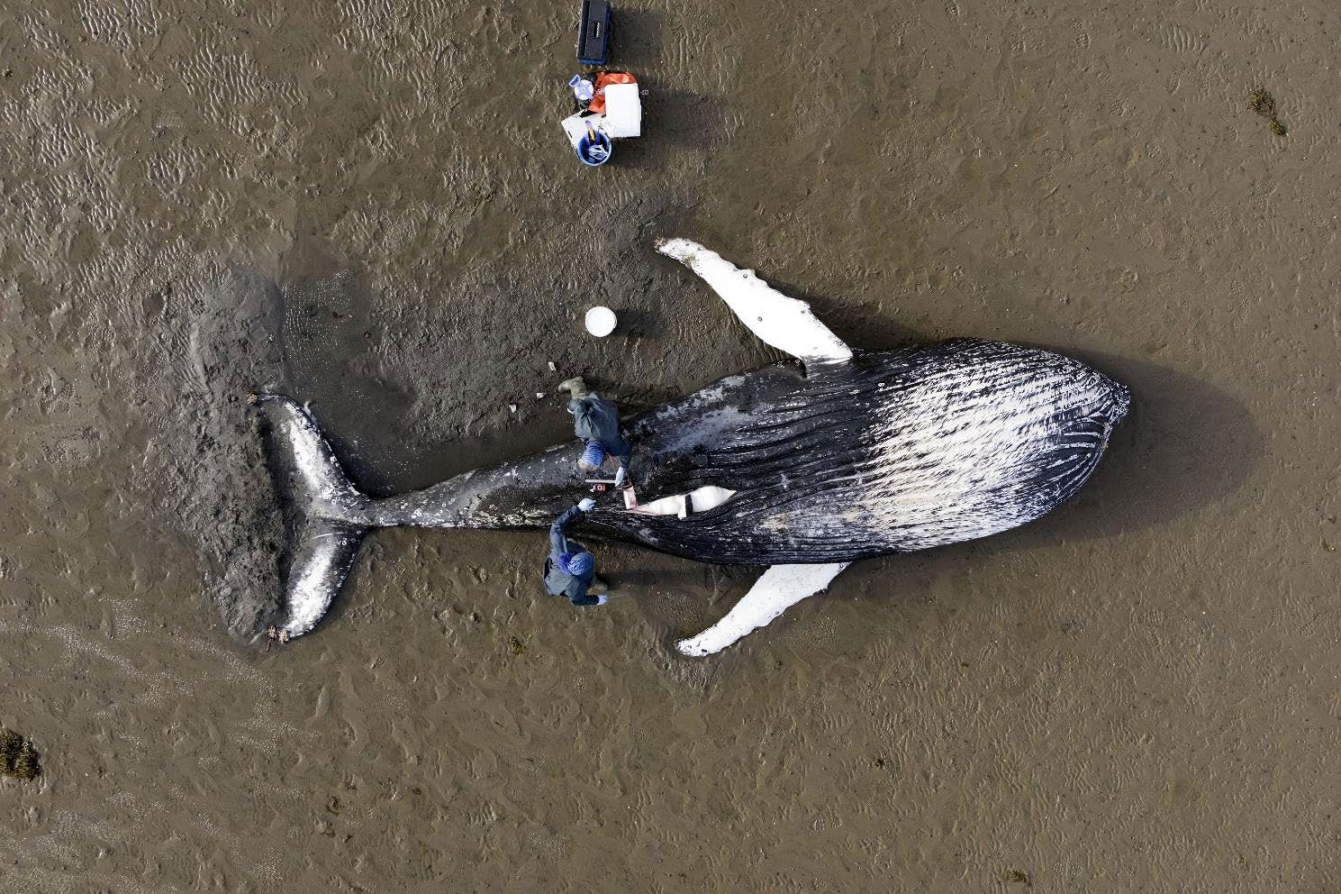
Researchers from the University of Glasgow’s Scottish Marine Animal Stranding Scheme conduct a necropsy of a stranded humpback whale. Attribution: Submitted by Professor Paul Thompson, photo captured by James Bunyan from Tracks Ecology

The runner-up for the ‘Research in action’ category captured a moment as researchers conduct a necropsy on a stranded humpback whale that, unfortunately, had passed away. Professor Paul Thompson from the University of Aberdeen in the UK, who submitted the photo taken by James Bunyan from Tracks Ecology explains, “Post whaling recovery of North Atlantic humpback whale populations has led to increases in sightings of this species in UK coastal waters, but this also raises the risk of entanglement in coastal waters. In May 2023, a young humpback whale became stranded in Loch Fleet National Nature Reserve in NE Scotland, drawing the attention of the scientific community. This site has been the focus of long-term photographic studies of harbour seals by the University of Aberdeen, most recently working in collaboration with Tracks Ecology to use UAV (unmanned aerial vehicle) photography to identify and measure individual seals from this population. Colleagues from the University of Glasgow’s Scottish Marine Animal Stranding Scheme conducted a necropsy of this whale, confirming drowning following entanglement was the most likely cause of death. This image was drawn from a series captured by UAV photography, documenting the necropsy and producing a 3-D model of the whale using photogrammetric techniques that were developed to study the size structure of the seal population.” This photo depicts researchers collaborating to further our understanding of the challenges faced by these magnificent creatures. (Fig. 3).

## Protecting our planet: best in category

**Fig. 4:**
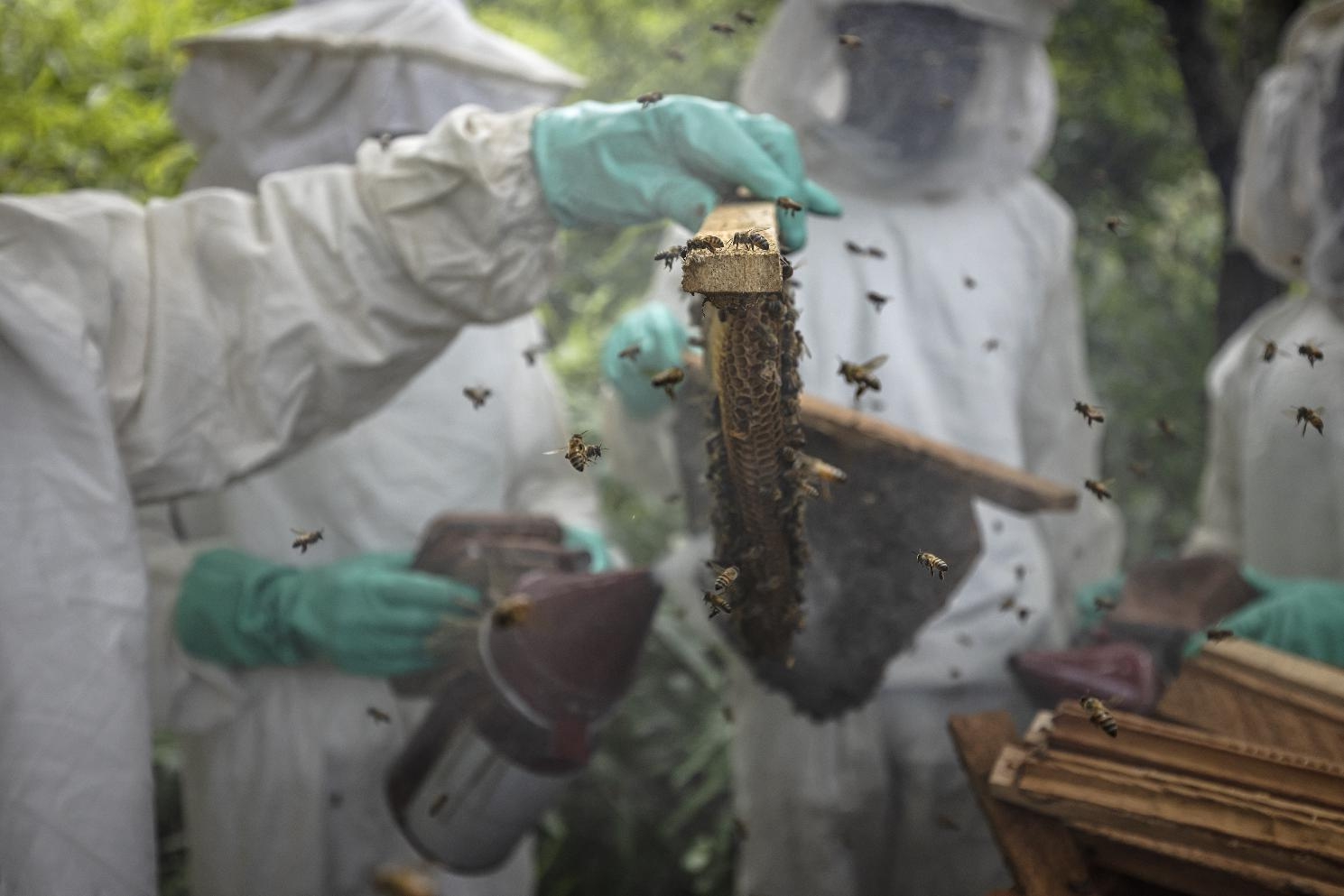
Sustainable beekeeping for chimpanzees. Attribution: Roberto García-Roa.

Roberto García-Roa, an evolutionary biologist and conservation photographer affiliated with the University of Lund in Sweden submitted the winning image of the ‘Protecting our planet’ category. The winning photograph beautifully captures the efforts of The Chimpanzee Conservation Center in Guinea to protect our planet and empower local communities. Roberto explains that, “their sustainable beekeeping project, launched in the surrounding villages of Faranah, showcases an inspiring solution to combat deforestation caused by traditional honey harvesting from wild bees. By cultivating their own honey, the locals avoid tree felling and increase production. What makes this initiative even more exceptional is that a portion of the profits generated is dedicated to the conservation of chimpanzees.” Senior Editorial Board Member Josef Settele comments, “This photo shows how very different aspects of wildlife conservation can be combined. Here, honey beekeeping helps create income and highlights the value of forests and the importance of the functioning of ecosystems to provide ecosystem services such as pollination and honey. Through the activities captured in the picture, the conservation of Chimpanzees – our close relatives – is a core result of community engagement. For me, this photo shows a great win-win situation and an excellent example of how one can contribute to protecting our planet.” (Fig. 4).

## Protecting our planet: runner-up

**Fig. 5:**
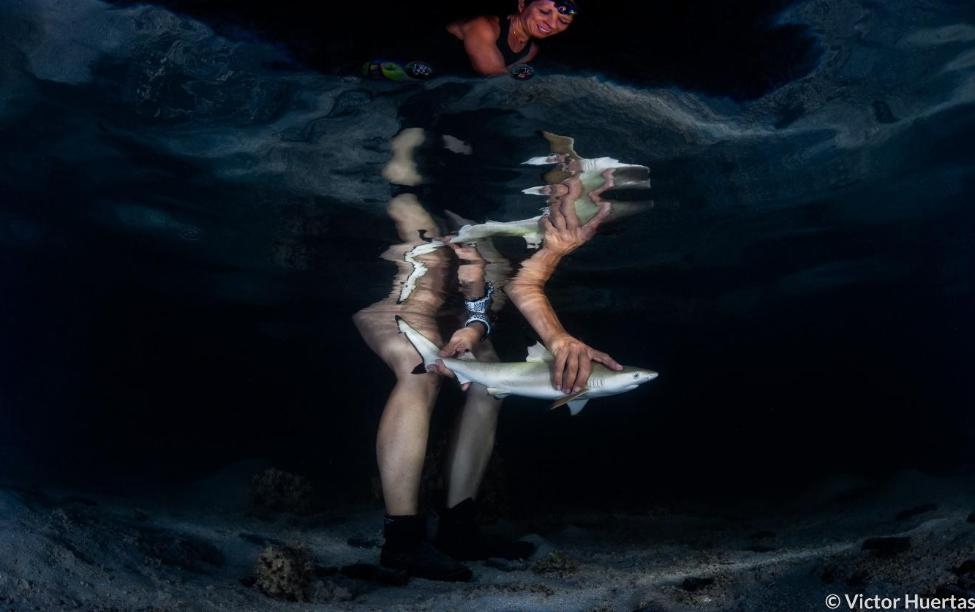
Protecting future generations of reef sharks. A researcher releases a new-born blacktip reef shark (*Carcharhinus melanopterus*) in Mo’orea, French Polynesia. Attribution: Victor Huertas

An image captured by Victor Huertas was also selected as the runner-up for the “Protecting our planet” category. The photo captures Professor Jodie Rummer, from James Cook University in Australia, releasing a newborn blacktip reef shark (Carcharhinus melanopterus) in Mo’orea, French Polynesia, after tagging it and collecting biometric data. Victor explains that “Professor Rummer leads Physioshark, a research team headquartered at James Cook University in Australia, that investigates the impact of climate change on the physiological performance of newborn sharks in tropical shark nurseries. These habitats typically occur in shallow waters and are therefore highly exposed to rising temperatures and lower oxygen concentrations. The Physioshark team is untangling the challenges newborn sharks face in such rapidly changing environmental conditions. Professor Rummer and her students have so far been able to show how despite the burden climate change is placing on the physiology of young sharks, these are displaying an exceptional resilience to these changes, giving scientists hope that they will be able to adjust to a warming ocean.” (Fig. 5).

## Plants and fungi: best in category

**Fig. 6:**
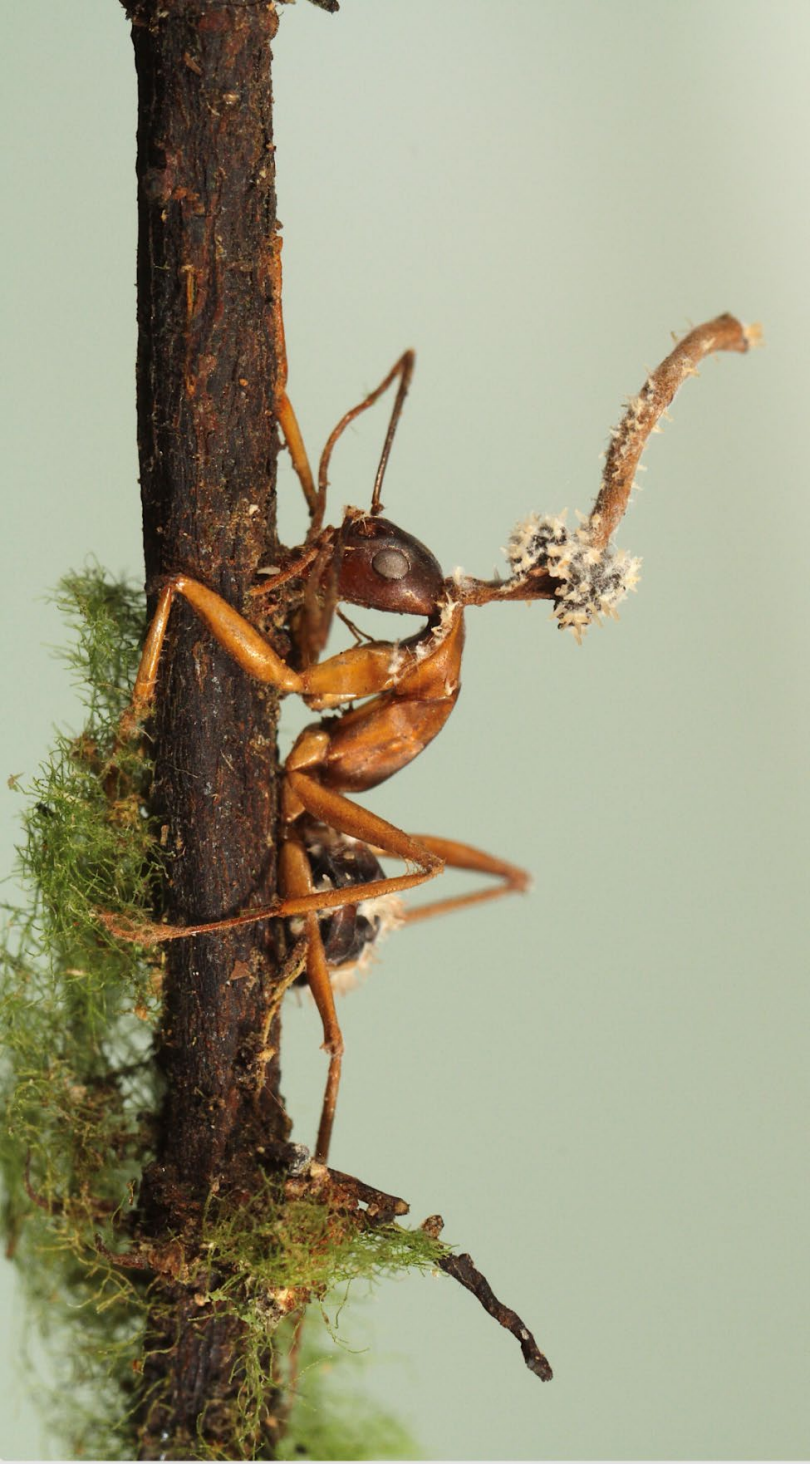
A mycoparasitic fungus parasitizing the fruiting body of a zombie-ant fungus. Attribution: João Araújo

João Araújo, a mycologist at the New York Botanical Garden, submitted the winning photo for the ‘Plants and fungi’ category. The image captures a fascinating scene of a fungus parasitizing the fruiting body of a zombie-ant fungus. Zombie-ant fungi possess the remarkable ability to manipulate the behaviour of their insect hosts, compelling them to migrate to a more favourable location for their growth. These incredible organisms infect various Camponotini ants in forests worldwide, from tropical to temperate regions. However, João’s photograph demonstrates that the life of this parasitic fungus is far from simple, depicting the complexity and intricacy of nature. João tells us, “The forests these fungi inhabit are also shared with mycoparasitic fungal lineages that can parasitize, consume and even castrate *Ophiocordyceps*. Only recently scientists have started to catalogue and describe these still unknown fungi that can kill other fungi [10]”. (Fig. 6).

## Plants and fungi: runner-up

**Fig. 7:**
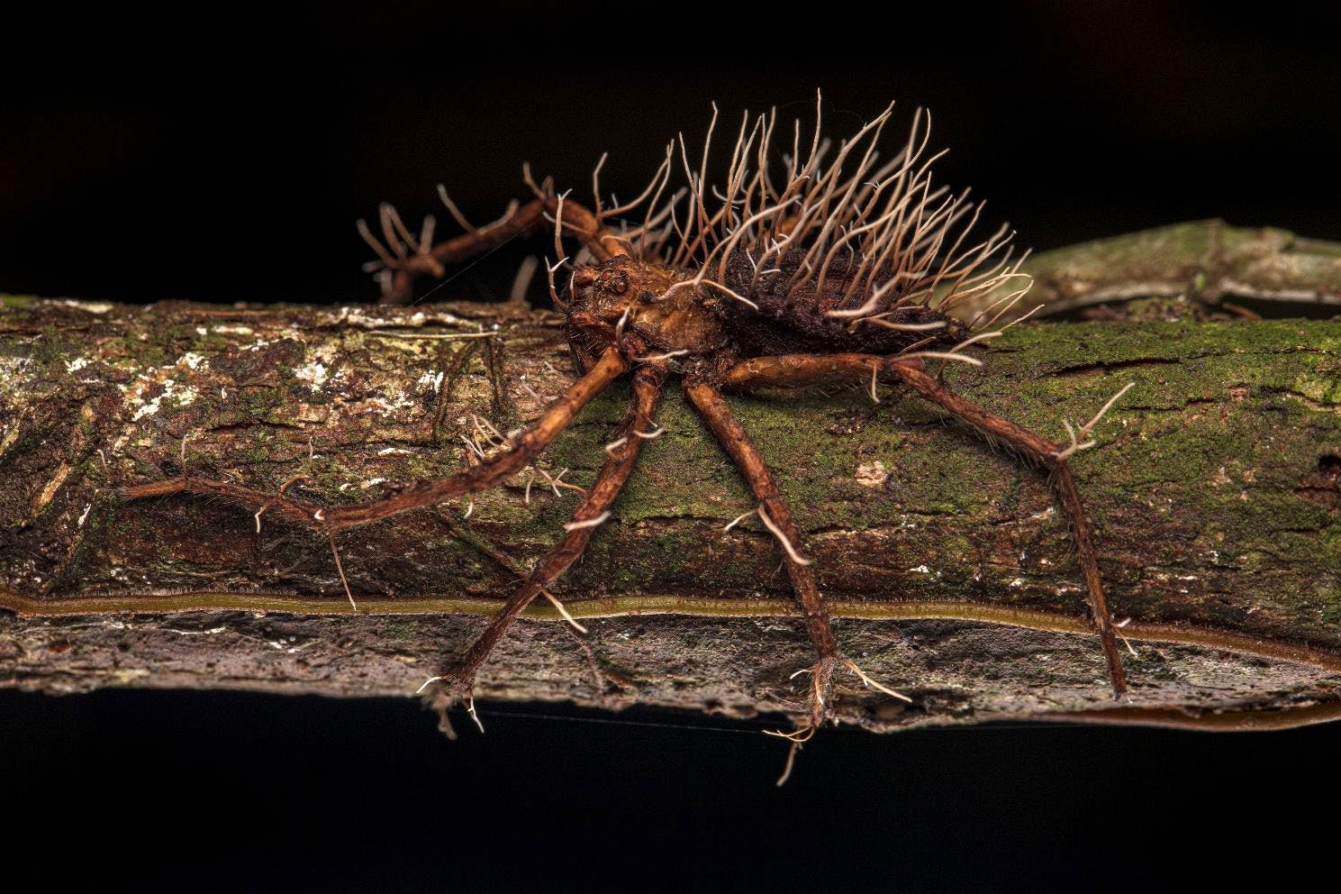
Defeated. A spider seemingly defeated by a parasitic fungus. Attribution: Roberto García-Roa.

Roberto García-Roa also submitted the runner-up for the ‘Plants and Fungi’ category. This unsettling yet mesmerizing image depicts a spider seemingly defeated by a parasitic fungus. Roberto explains “While it is not uncommon to encounter insects parasitised by “zombie” fungi in the wild, it is a rarity to witness large spiders succumbing to these fungal conquerors. In the jungle, near a stream, lies the remains of a conquest shaped by thousands of years of evolution.” Senior Editorial Board Member Luke Jacobus comments “The image provides an opportunity to expand awareness and understanding of complex and unfamiliar interactions between organisms.” (Fig. 7).

## Paleoecology: best in category

**Fig. 8:**
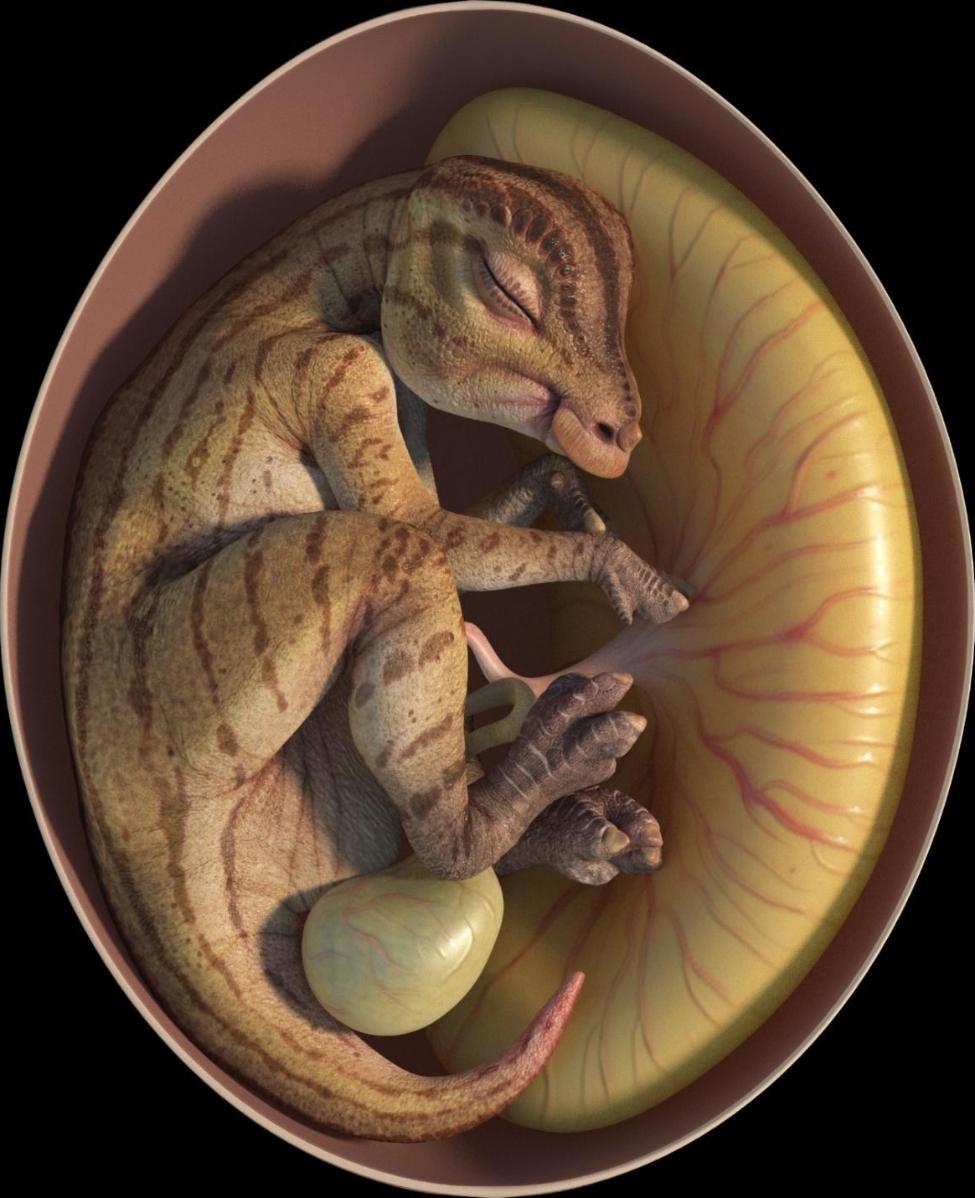
A peek inside a hadrosaur egg. Attribution: Submitted by Jordan Mallon. Restoration by Wenyu Ren

The winning image of the ‘Paleoecology’ category, submitted by Jordan Mallon from the Canadian Museum of Nature, has captivated our judges and highlighted the incredible work a group of trans-Pacific palaeontologists completed remotely during the COVID-19 pandemic [11]. Jordan’s submission showcases the team’s collaborative efforts in describing a remarkable discovery: a pair of hadrosauroid dinosaur eggs and embryos from China’s Upper Cretaceous red beds, dating back approximately 72 to 66 million years ago. Jordan comments, “The relatively small size of the eggs, and the unspecialized nature of the dinosaur embryos inside, suggest that the earliest hadrosaurs laid small eggs and hatched altricial young. More derived hadrosaurs eventually laid eggs nearly four times larger by volume and hatched correspondingly larger young. This digital image depicts an example of a ‘primitive’ hadrosaur developing within the safety of its small egg expertly crafted by Wenyu Ren.“ This image reminds us of the amazing wealth of information locked within fossils. (Fig. 8).

## Paleoecology: runner-up

**Fig. 9:**
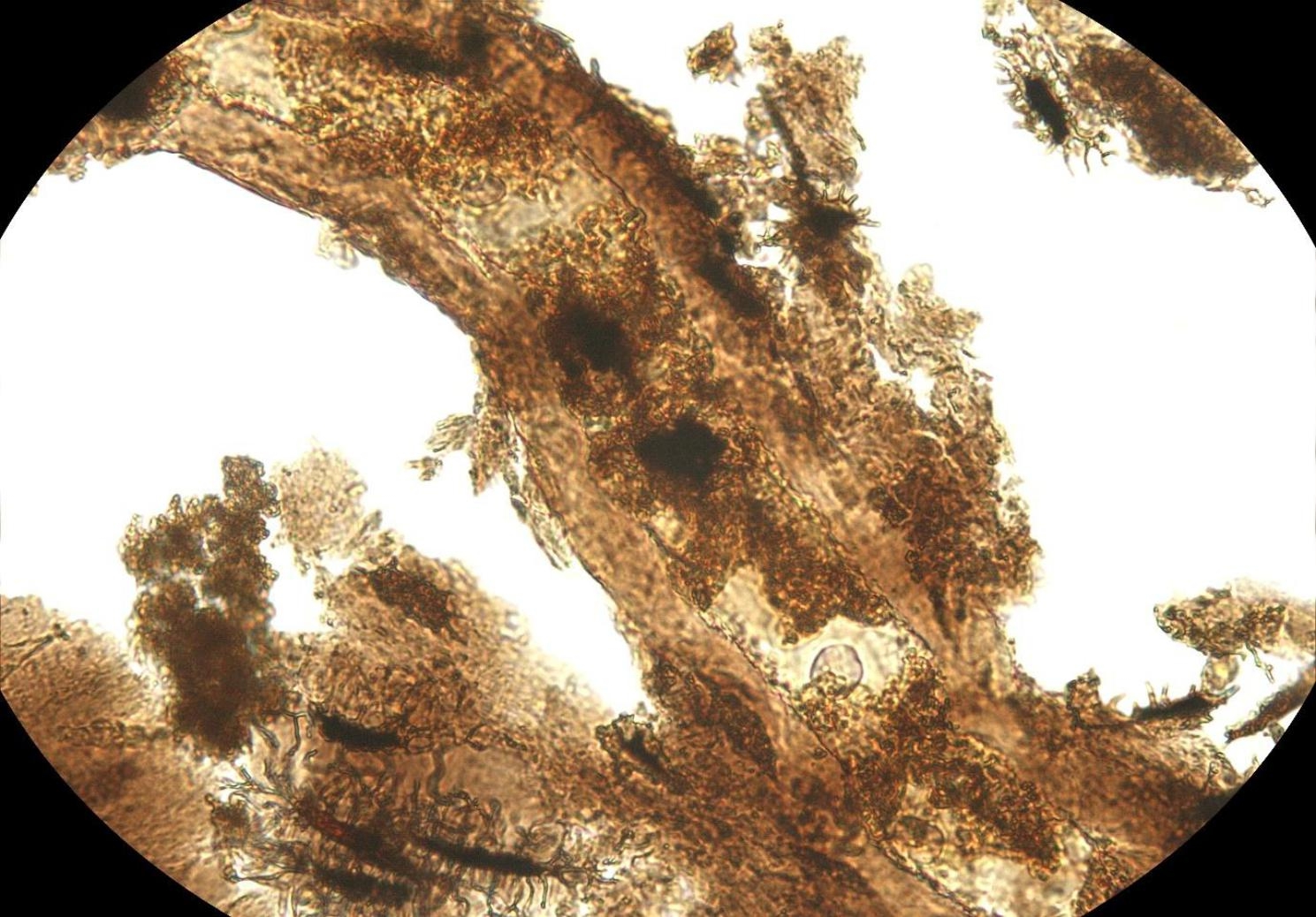
Paradoxical preservation. Microscopy reveals an extracted diplodocid dinosaur blood vessel. Attribution: Dr Jasmina Wiemann

Dr Jasmina Wiemann, a molecular paleobiologist from the Negaunee Integrative Research Center, Field Museum of Natural History, USA, entered the runner-up for the ‘Paleoecology’ category. Through her microscope, Jasmina provides a view of an extracted diplodocid dinosaur blood vessel surrounded by preserved extracellular matrix containing remnants of cells that are approximately 150 million years old. Jasmina explains, “Once considered paradoxical, the preservation of fragile soft tissues is now known to be the result of the chemical transformation of original proteins, lipids, and sugars occurring during fossilization - allowing such fragile evidence of past life to survive over millions of years!“. The field of molecular paleobiology is important for reconstructing the physiology, relationships and behaviours of long-extinct species. (Fig. 9).

## Conclusions

We thank everyone who participated in this year’s BMC Ecology and Evolution image competition. The competition produced a spectacular collection of images showcasing nature’s wonders and the remarkable work conducted by ecologists, evolutionary biologists and palaeontologists worldwide. As we bid farewell to this year’s competition, we eagerly anticipate next year’s event to continue our photographic celebration of ecology and evolutionary biology.

Figures 1, 2, 3, 4, 5, 6, 7, 8 and 9 in this Editorial are released under a Creative Commons Attribution License (CC BY) to ensure credit with proper attribution [12]. If you wish to re-distribute or re-use any Figs. 1, 2, 3, 4, 5, 6, 7, 8 and 9 published in this editorial, please credit individual winners as the image licensee.
